# Frequency of the anti-glutamic acid decarboxylase immunological marker in patients with diabetes duration longer than three years in southern Brazil

**DOI:** 10.1590/S1516-31802011000300002

**Published:** 2011-05-05

**Authors:** Marina Carolina Moreira, Gustavo Müller Lara, Rafael Linden, Luciane Rosa Feksa, Rejane Giacomelli Tavares, Sabrina Esteves de Matos Almeida, Daiane Bolzan Berlese

**Affiliations:** I BSc in Biomedicine, Universidade Feevale, Novo Hamburgo, Rio Grande do Sul, Brazil.; II MSc. Teacher responsible for the Immunology Laboratory Course for Undergraduate Biomedicine, Universidade Feevale, Novo Hamburgo, Rio Grande do Sul, Brazil.; III PhD. Professor in the Institute of Health Sciences and Researcher in the Bioanalysis Research Group, Universidade Feevale, Novo Hamburgo, Rio Grande do Sul, Brazil.; IV PhD. Adjunct professor and Researcher in the Bioanalysis Research Group, Universidade Feevale, Novo Hamburgo, Rio Grande do Sul, Brazil.

**Keywords:** Antibodies, Diabetes mellitus, type 1, Autoimmunity, Chronic disease, Incidence, Anticorpos, Diabetes mellitus tipo 1, Autoimunidade, Doença crônica, Incidência

## Abstract

**CONTEXT AND OBJECTIVE:**

The anti-GAD (glutamic acid decarboxylase) antibody is considered to be an important marker for type 1 diabetes mellitus (DM1), with frequency that varies depending on the population studied and the duration of the disease. Therefore, the aim of this study was to determine the frequency of this autoantibody in a group of patients in southern Brazil with DM1 that had been diagnosed more than three years previously.

**DESIGN AND SETTING:**

Analytical cross-sectional study with a control group conducted at the Biomedicine Laboratory of Universidade Feevale.

**METHODS:**

This study was conducted between June 2007 and December 2008, and 109 individuals were enrolled during this period. Fifty-eight were DM1 patients and 51 were individuals free from DM1 and without any history of diabetes, who constituted the control group.

**RESULTS:**

In the DM1 group, the mean age was 27 ± 1.7 years and 50% were men. The mean fasting blood glucose in the DM1 group was 208 ± 15 mg/dl and mean HbA1c (glycosylated hemoglobin) was 8.7 ± 0.25%. In the control group, the mean fasting blood glucose and HbA1c were 82 mg/dl and 5.0% respectively. Thirty-seven individuals with DM1 (63.8%) were positive for anti-GAD, and this proportion was significantly larger than in the control group.

**CONCLUSIONS:**

These results show the high prevalence of anti-GAD in the population of diabetic patientcous in southern Brazil, thus indicating that the antibody was still present a long time after the disease had been diagnosed.

## RESUMO

**CONTEXTO E OBJETIVO:**

O anticorpo anti-decarboxilase do ácido glutâmico (anti-GAD) é considerado um importante marcador no diabetes *mellitus* tipo 1 (DM1), cuja frequência varia segundo a população estudada e o tempo de duração da doença. Assim, o objetivo deste estudo foi determinar a frequência deste auto-anticorpo em um grupo de pacientes localizados no Sul do Brasil com mais de três anos de diagnóstico de DM1.

**TIPO DE ESTUDO E LOCAL:**

Estudo transversal analítico com grupo controle, realizado no Laboratório de Biomedicina da Universidade Feevale.

**MÉTODOS:**

Este estudo foi realizado no período de Junho de 2007 a Dezembro de 2008, em que 109 indivíduos foram incluídos, sendo 58 destes com DM1 e 51 indivíduos sem DM1 e sem antecedentes de diabetes, que constituíram o grupo controle.

**RESULTADOS:**

No grupo DM1, a idade média foi 27 ± 1,7 anos e 50% eram homens. A média da glicemia de jejum no grupo DM1 foi 208 ± 15 mg/dL e a HbA1c média foi 8,7 ± 0.25%. No grupo controle a glicemia de jejum média e a HbA1c (hemoglobina glicosilada) foram 82 mg/dL e 5,0%, respectivamente. O anti-GAD foi positivo em 37 (63,8%) indivíduos com DM1, valores significativamente maiores quando comparados com os do grupo controle.

**CONCLUSÕES:**

Estes resultados mostram a alta prevalência do anti-GAD na população de pacientes diabéticos da região Sul do Brasil, indicando que o anticorpo está presente após um longo período de diagnóstico da doença.

## INTRODUCTION

Diabetes mellitus type 1 (DM1) is an organ-specific chronic autoimmune disease that accounts for 5-10% of all cases of diabetes and is characterized by progressive immunomediated destruction of pancreatic beta-cells, thereby resulting in insufficient insulin production.^1^ The immunogenetic and environmental factors associated with the onset and maintenance of the pancreatic lesions observed in DM1 are not yet fully understood.^2^

Cell destruction proceeds at an intense and rapid rate when the onset is during childhood or adolescence, which leads to early and constant treatment with insulin, whereas when it emerges in adulthood, the process is slower and patients may retain residual beta-cell function for some years after diagnosis.^3,4^

The autoimmune nature of DM1 is caused by the presence of circulating autoantibodies, including islet cell autoantibodies (ICA), insulin autoantibodies (IAA) and anti-glutamic acid decarboxylase (GAD).^5^ These markers, alone or in combination, are found in 85-90% of individuals with a recent diagnosis of DM1.^3^ Fifty percent of adult patients who are anti-GAD-positive at diagnosis exhibit a clear insulin deficiency within 10 years, compared with just 3% of anti-GAD-negative patients.^6^ Anti-GAD tends to be the first marker to appear, years before disease onset, with a diagnostic sensitivity of 75-85% and specificity of 98-99%.^7^ These antibodies can be assayed in clinical practice in situations in which diabetes cases cannot be classified on clinical presentation alone.^8^

The data available on the frequency of anti-GAD in the population of southern Brazil are still limited, and additional studies to assess the frequency, taking ethnic differences in the Brazilian population into account, are needed.

## OBJECTIVE

Data on the frequency of these autoantibodies in the population is important for achieving better understanding and diagnoses of DM1. Therefore, the objective of this study was to determine the frequency of anti-GAD autoantibodies in a group of patients in southern Brazil with DM1 that had been diagnosed at least three years previously.

## MATERIALS AND METHODS

This was an analytical cross-sectional study with a control group that was conducted between June 2007 and December 2008 at the Biomedicine Laboratory of Universidade Feevale, which is located in Novo Hamburgo, a city within the metropolitan area of Porto Alegre. The study population comprised 109 individuals: 58 were DM1 patients and 51 were individuals without DM1 (control group). The DM1 patients had diabetes diagnoses that had been confirmed in accordance with World Health Organization criteria more than three years prior to enrollment, and they were on a continuous insulin regimen. Only patients who had no complications relating to diabetes were included.

Patients registered within the Brazilian National Health System (SUS) in the city of Novo Hamburgo were invited to participate in the study. The sample was selected by telephone (random dialing) and advertising in the local newspaper. Those who agreed to participate in the study were given appointments to attend the Biomedicine Laboratory of Universidade Feevale. Data collection always took place in the mornings.

The control group consisted of subjects without any history of diabetes, and with normal fasting glucose. The control group was formed by residents of the city of Novo Hamburgo who had registered to participate and who met the inclusion criteria. The recruitment was done through advertisements in local newspapers.

A sample size calculation showed that it would take at least 57 DM1 patients to identify an appropriate amount with 95% confidence and sampling error of 5%. This number was assigned by taking into consideration that the population of Novo Hamburgo is about 250,000 inhabitants and at least 5% of the population has DM1.

All subjects were invited to take part in this study and, if they accepted, they signed a free and informed consent statement and filled out questionnaires to provide data on age, sex, date of DM1 diagnosis and insulin treatment. The patients were included only if they had been diagnosed more than three years before the time of this study and less than five years before this time. The study was approved by the Ethics Committee of Universidade Feevale (protocol number 2.08.01.07.571).

In addition to assaying anti-GAD, fasting blood glucose was measured only once, in the morning, in all participants and a whole blood sample was taken in order to measure glycosylated hemoglobin (HbA1c) to assess glycemic control. These measurements were not used to establish a correlation between HbA1c and metabolic control, but were used with the intention of characterizing the sample.

### Assaying anti-GAD, HbA1c and fasting blood glucose

Blood samples were collected into two tubes per subject, one containing EDTA (ethylenediaminetetraacetic acid) for the HbA1c assay, and the other without anticoagulant to provide serum for the anti-GAD and fasting blood glucose assays. Both HbA1c and fasting blood glucose were assayed immediately after collection, while serum for the anti-GAD assay was aliquoted and frozen at −70 ºC for later analysis.

The HbA1c assay was measured using a commercial kit (Doles, Doles Reagentes e Equipamentos de Laboratório Ltda, Goiânia, Brazil). The blood glucose was measured using a commercial kit (Roche Diagnostics, Mannheim, Germany).

Anti-GAD was assayed using ELISA (enzyme-linked immunosorbent assay) by means of a commercial autoantibody (GAD65 ELISA kit; RSR Ltd., Cardiff, United Kingdom). Subjects were defined as anti-GAD-positive if their titers were ≥ 5 U/ml. All analyses were conducted in duplicate.

### Statistical analysis

The data on fasting blood glucose and HbA1c were presented as means and standard errors. The data on age, sex and anti-GAD titers were analyzed using the chi-square test. The *t* test for two samples assuming variance was used to compare the mean ages of the two groups. Data were expressed as the mean ± standard error of the mean.

## RESULTS

### Demographic and clinical data

The study population included 109 individuals, split between a control group (51 subjects) and DM1 patients (58 subjects). In the DM1 group, the average age was 27 ± 1.7 years and 50% were men. There was no statistical difference between the patients and controls in terms of age (P > 0.05).

The mean fasting blood glucose level in the DM1 group was 208 ± 15 mg/dl and the mean HbA1c was 8.7 ± 0.25%. In the control group, the mean fasting blood glucose and HbA1c were 82 mg/dl and 5.0% respectively (Table 1).

**Table 1.** Epidemiological and clinical characteristics of patients and controls

	DM1	Control group
Sex (M/F), mean	29/29	38/13
Age (years), mean ± standard error*	27 ± 1.7	27 ± 1.4
Fasting blood glucose (mean ± standard error)†	208 ± 15 mg/dl	82 ± 2.3 mg/dl
HbA1c (mean ± standard error)†	8.7% ± 0.25	5% ± 0.06

* t test for two samples assuming variance;

† chi-square test.

DM1 = type 1 diabetes mellitus; HbA1c = glycosylated hemoglobin.

### Frequency of anti-GAD

Thirty-seven DM1 patients (63.8%) were positive for anti-GAD, while this was not present, or was within normal limits (< 5 U/ml), in 21 (36.2%) of the control subjects (P < 0.05). All of the individuals in the control group were within the reference limits for anti-GAD-negative results (Table 2).

**Table 2.** Frequency of anti-GAD (glutamic acid decarboxylase) autoantibodies in patients with type 1 diabetes mellitus (DM1) diagnosed more than three years previously

	GAD-positive	GAD-negative	P*
DM1	63.8% (37/58)	36.2% (21/58)	< 0.05
Control group	0 (0/51)	100% (51/51)	-

* Chi-square test.

## DISCUSSION

DM1 is an organ-specific chronic autoimmune disease with highly complex pathogenicity. Early emergence of certain autoantibodies such as pancreas islet cell autoantibodies, insulin autoantibodies and anti-glutamic acid decarboxylase is an important characteristic of this pathological condition.^9^

Several studies have reported that antibody titers in DM1 patients tend to reduce as the years pass, and in particular, anti-GAD titers.^10^ However, in the present study, we observed that 63.8% of a study population that had been diagnosed with DM1 more than three years previously and was on insulin therapy was anti-GAD-positive.

Other studies have also found that in patients with late-onset autoimmune diabetes of adulthood, not only anti-GAD, but also ICA and protein tyrosine phosphatase-like protein antibodies (anti-IA2) remain after diagnosis, for a longer period than in children with DM1.^11,12^ It is possible that anti-GAD positivity is related to some residual beta cell function, although we did not investigate this in the present study.

It has been shown that age at diagnosis does not affect anti-GAD positivity.^13^ However, we cannot rule out the possibility that the frequency of anti-GAD may vary between different ethnic groups, or that there may be differences within the Brazilian population. This study was conducted in southern Brazil, where the population is characterized by major European colonization (German and Italian in particular).

The parameters of fasting blood glucose and glycosylated hemoglobin were also assessed in this study. The mean fasting blood glucose level in the DM1 group was 208 mg/dl. The glycosylated hemoglobin level in the DM1 group was 8.7%. These data not only confirm the DM1 diagnosis, but also are also highly worrying in this population, with regard to metabolic control. It has been shown that abnormal fasting blood glucose levels increase the cardiac risk in patients with DM1.^14^ Glycosylated hemoglobin is the principal test for monitoring chronic complications of DM1, and these chronic complications bear the main responsibility for mortality and morbidity among diabetic patients. Glycosylated hemoglobin levels above 7% are associated with a progressively greater risk of chronic complications.^15^

Autoimmune diabetes is common among Brazilian adults and children, and its clinical manifestations may be similar to those of other known types of diabetes. Therefore, assaying autoantibodies may be an important ally for differential diagnosis and may aid clinicians with regard to treatment principles. Nevertheless, further studies are needed in order to elucidate this issue and gain better understanding of the etiology of DM1, and also to discover why these antibodies remain present for years after diagnosis. Effective metabolic control measures need to be taken in order to avoid the complications of diabetes and improve these patients’ quality of life.

## CONCLUSION

These results show the high prevalence of anti-GAD in the population of diabetic patients in southern Brazil, thus indicating that the antibody was still present a long time after the disease had been diagnosed. However, further studies are needed to elucidate the relationship between antibody and DM1.
